# Effect of respiratory muscle training on diaphragm function in stroke patients: a systematic review and meta-analysis

**DOI:** 10.3389/fmed.2025.1694356

**Published:** 2026-01-20

**Authors:** Jianglong Chen, Yu Yin, Jianyang Xu, Peiyuan Lv, Weibo Li

**Affiliations:** 1 Department of Rehabilitation, Hebei General Hospital, Shijiazhuang, China; 2 Department of Neurology, Hebei General Hospital, Shijiazhuang, China; 3 Department of Gastrointestinal Surgery, The Second Hospital of Hebei Medical University, Shijiazhuang, China

**Keywords:** diaphragm function, meta-analysis, post-stroke, respiratory muscle training, stroke

## Abstract

**Objective:**

This meta-analysis aimed to assess the effectiveness of respiratory muscle training (RMT) in restoring diaphragm function after a stroke.

**Methods:**

We conducted a comprehensive search for studies investigating the impact of RMT on diaphragm function in post-stroke patients that were published in the China National Knowledge Infrastructure, Wanfang Data, PubMed, Embase, Cochrane Library, Web of Science, Physiotherapy Evidence Database and ClinicalTrials.gov databases between inception and April 2023. Six reviewers independently screened eligible studies, extracted data and assessed methodological quality. The results were analysed using mean differences (MDs) with 95% confidence intervals (CIs), and heterogeneity was assessed using the chi-squared test and *I*^2^ statistic.

**Results:**

This meta-analysis included 6 studies comprising 246 patients, with methodological quality ranging from poor to excellent. We observed significant differences in diaphragm mobility on the affected side after a stroke (MD = 1.32, 95% CI: 0.96–1.67; *p* < 0.00001) as well as in affected side diaphragm thickness (DT) at inspiration (A-DTI) (MD = 0.08, 95% CI: 0.03–0.14; *p* = 0.002), affected side DT at expiration (A-DTE) (MD = 0.01, 95% CI: 0.00–0.02; *p* = 0.13), non-A-DTI (MD = 0.03, 95% CI: 0.02–0.04; *p* < 0.00001), non-A-DTE (MD = 0.01, 95% CI: −0.01–0.02; *p* = 0.56) and affected side diaphragm thickening fraction (DTF) (MD = 47.32, 95% CI: 11.04–83.60; *p* = 0.01) non-affected DTF (MD = 15.47, 95% CI: −12.19–43.13; p = 0.27).

**Conclusion:**

Respiratory muscle training can enhance diaphragm function in post-stroke patients, encompassing improvements in diaphragm mobility, thickness and thickening fraction, particularly focusing on the affected side diaphragmatic function.

**Systematic review registration:**

Trial registration CRD42022371157, available from https://www.crd.york.ac.uk/PROSPERO/view/CRD42022371157.

## 1 Introduction

Stroke stands as the foremost cause of mortality and disability among adults, marked by elevated rates of morbidity, disability, mortality and recurrence (1). In individuals affected by stroke, the central nervous system injury and other factors not only impair voluntary movements and coordination of trunk muscles, leading to abnormal posture and muscle tone, but also accentuate the asymmetry between the paralysed and non-paralysed sides of the diaphragm, thereby causing diaphragm dysfunction (2). The precise mechanism remains a matter of debate, potentially involving altered diaphragm function due to corticospinal tract injury and diaphragmatic autonomic dysfunction due to medullary spinal tract injury. Nonetheless, other research has suggested that stroke-related diaphragm involvement may be linked to disruptions in corticodiaphragmatic pathways (3). Patients experiencing diaphragmatic dysfunction following a stroke exhibit restrictive ventilatory impairment, characterised by a substantial reduction in maximal spontaneous ventilation, maximal respiratory pressure, diminished diaphragmatic movement amplitude during breathing activities, diminished and uneven contractile capacity and a corresponding reduction in DT (4). Studies have indicated that the prevalence of diaphragmatic dysfunction within the first 48 h of supratentorial ischaemic stroke is notably high, at 51.7% (3). Furthermore, the incidence of diaphragmatic dysfunction in stroke patients during recovery stands at 46.67% (5). In one investigation, 40% of stroke patients displayed reduced diaphragmatic movement on the hemiplegic side of their body (6). Decreased diaphragmatic function significantly correlates with motor, respiratory and balance impairments in stroke patients, potentially prolonging the recovery process and straining medical resources (5, 7–9).

Respiratory muscle training (RMT) encompasses both conventional respiratory muscle training, including techniques such as abdominal breathing and chest expansion training, and threshold loading training utilising specialised devices. Threshold loading training equipment offers repetitive breathing exercises employing flow-dependent resistance or pressure thresholds, primarily targeting the diaphragm as the primary inhalation muscle (10). The diaphragm, responsible for 70% of ventilatory activity, can be strengthened by modulating resistance intensity, resulting in adaptive changes in muscle fibre structure under overload conditions, akin to other skeletal muscles (11).

Previous investigations have established the effectiveness of RMT in improving pulmonary and diaphragmatic function in stroke patients (12–14) and have affirmed a substantial association between pulmonary function and diaphragmatic function (8, 9). Many previous systematic reviews on the effect of RMT on stroke patients have used indicators such as maximal inspiratory pressure (MIP) and maximal expiratory pressure (MEP); however, the measurement of MIP and MEP may be affected by the ability of the mouth to seal, causing imprecision of the results. Few studies have examined changes in the diaphragm; one previous meta-analysis (15) included only 3 studies on the effect of RMT on DT after stroke, and the meta-analysis only analysed the effect of RMT on DT and did not address diaphragm mobility or the diaphragm thickening fraction (DTF). Consequently, it is imperative to conduct a meta-analysis of randomised controlled trials to assess the efficacy of RMT in enhancing diaphragm function among individuals recovering from stroke.

## 2 Materials and methods

This study was registered with the International Prospective Register of Systematic Reviews, under the registration number CRD42022371157. The research was conducted in accordance with the Preferred Reporting Items for Systematic Reviews and Meta-Analyses guidelines (16).

### 2.1 Search strategy

Two researchers independently conducted searches in the China National Knowledge Infrastructure, Wanfang Data, PubMed, Embase, Physiotherapy Evidence Database (PEDro), Web of Science, ClinicalTrials.gov and the Cochrane Library databases, covering the period between inception and April 2023, with no language restrictions. Any disagreements were resolved through consensus or consultation with a third investigator. The search involved the use of specific keywords and Medical Subject Headings terms, including ‘stroke’, ‘cerebral stroke’, ‘cerebrovascular apoplexy’, ‘breathing exercises’, ‘respiratory muscle training’ and ‘inspiratory muscle training’. Additional relevant studies were identified by reviewing the reference lists of included articles and previous systematic reviews. A detailed search strategy is available in Supplementary Appendix 1.

### 2.2 Study selection and data extraction

Two reviewers independently performed study selection and data extraction. Any disagreements were resolved through consensus, and if disputes persisted, a third reviewer was consulted for resolution. The inclusion criteria were based on a randomised controlled trial (RCT) analysing the efficacy of RMT in enhancing diaphragm function in stroke patients and were as follows: (1) stroke (haemorrhagic or ischaemic), regardless of patient gender, age or time since stroke onset; (2) intervention involving inspiratory muscle training (IMT), expiratory muscle training (EMT) or a combination of both; (3) control group receiving no respiratory training or sham RMT without any resistance; and (4) outcome measures including affected side DT at inspiration (A-DTI), affected side DT at expiration (A-DTE), non-A-DTE (NA-DTE), non-A-DTI (NA-DTI), affected side DTF (A-DTF) non-A-DTF (NA-DTF) and diaphragm mobility on the affected side (A-DM).

The exclusion criteria were as follows: (1) studies lacking reported outcome variables; (2) non-RCTs; or (3) insufficient data.

For each included study, the following data were extracted: study details (authors, publication year and country), study design, participant characteristics (gender and age), sample size, intervention specifics (e.g., intervention content, duration, frequency, training duration and devices used), administration of the control group and outcome measures.

### 2.3 Risk of bias assessment

Two reviewers independently assessed the risk of bias using the Cochrane Risk of Bias Tool (17). This tool evaluates various categories, including random allocation, concealed allocation, blinding of participants and personnel, detection bias, attrition bias and reporting bias. The results for each category were categorised as high, low or unclear. In case of any disagreements during this process, a consensus was reached through discussion among the reviewers. Additionally, methodological quality was independently evaluated by the two reviewers using the PEDro scale (18). The PEDro scale is specifically designed for assessing the quality of physical therapy trials and consists of 11 items that assess internal validity factors, such as random and concealed allocation, baseline similarity, blinding and intention-to-treat analysis, as well as statistical reporting. The total PEDro score ranges from 0 to 10 points, excluding the first item on eligibility criteria. A higher score reflects better study quality (19). In instances where discrepancies arose in the evaluation, the final decision was made either through discussion or by consulting a third researcher.

### 2.4 Statistical analysis

Statistical analysis was conducted using Review Manager 5.4 software. As all results involved continuous data, the pooled effect size was expressed using the mean difference (MD) and 95% confidence interval (CI). Statistical heterogeneity was assessed using the *I*^2^ statistic. If *p* > 0.1 and *I*^2^ < 50%, indicating minimal heterogeneity among studies, a fixed-effects model was employed for meta-analysis. Conversely, if *p* ≤ 0.1 and/or *I*^2^ ≥ 50%, suggesting significant heterogeneity among studies, a random-effects model was utilised for meta-analysis. Sensitivity analyses were performed to identify potential sources of heterogeneity and to assess the robustness of the results by systematically excluding 1 study at a time. Subgroup analyses were conducted based on intervention duration using Review Manager 5.4 software. Notably, a publication bias analysis was not conducted due to the inclusion of <10 studies.

All statistical analyses, including the model selection and the additional analyses performed in response to reviewer comments, were reviewed and validated by an independent biostatistician to ensure methodological rigour.

## 3 Results

### 3.1 Study selection

**Figure 1:**
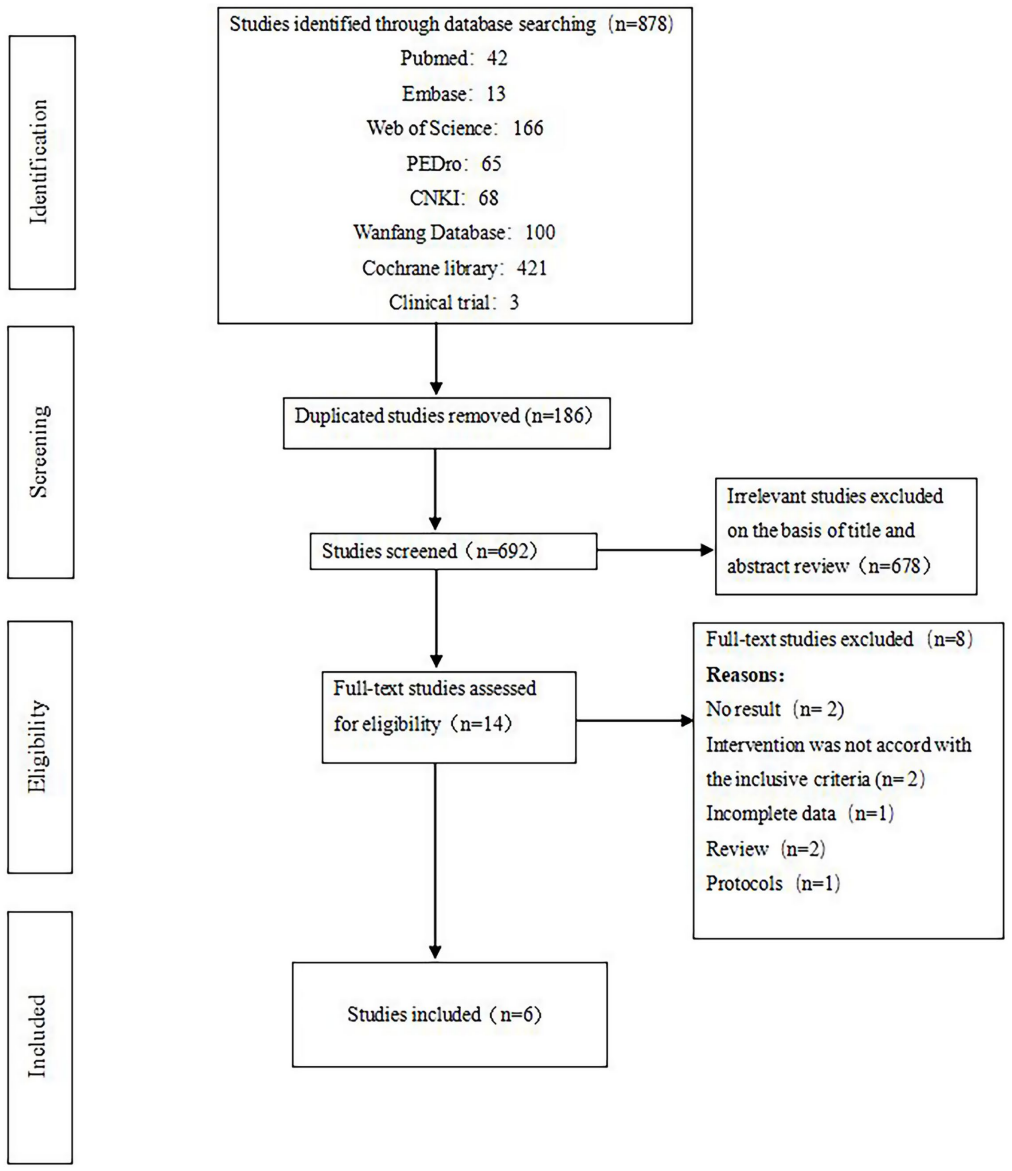
PRISMA flow chart.

The initial search strategy yielded a total of 878 studies. After removing duplicates, 692 unique studies remained. Following the screening of titles and abstracts, 678 studies were excluded as they did not meet the inclusion criteria, resulting in 14 relevant studies. After a thorough examination of the full texts, 6 RCT studies (9, 11, 20–23) were ultimately included in this meta-analysis. A detailed flowchart illustrating the selection process is presented in Figure 1.

### 3.2 Study characteristics

The 6 included RCT studies involved a total of 246 participants and were conducted in Korea, Turkey and China. These studies were published between 2013 and 2023. Participants’ average ages ranged from 52 to 66 years, and the time since stroke varied from 40 days to 11 months. All of the included studies utilised RMT, with 2 studies (9, 20) employing IMT and EMT and 4 studies (11, 21–23) solely focusing on IMT. Five studies (11, 20–23) employed a threshold device for respiratory muscle training, with all starting training at 30% of MIP. In 4 of these studies (20–23), the duration of each training session was 20 min, whereas in the other study (11), the training regimen was determined by the number of training sessions. In 1 study (9), therapists used an incentive spirometer in conjunction with a breathing exercise technique for respiratory muscle training. The intervention frequency was five times per week in 4 studies (9, 11, 21, 22) and three times per week in 2 studies (20, 23), with interventions lasting 4–6 weeks. In 5 studies (9, 11, 21–23), both the experimental and control groups received traditional stroke rehabilitation protocols. In another study (20), both the control and experimental groups received trunk stabilisation exercises in addition to the traditional stroke rehabilitation protocol. Various devices were used in the RMT training, including the Threshold PEP, Threshold IMT-Philips Respironics, Andover, MA, PowerBreath-K5 and Incentive spirometer. Among the included studies, 2 assessed A-DM, 5 assessed A-DTI and A-DTE, 4 assessed NA-DTI and NA-DTE, 4 assessed NA-DTF and only 1 assessed A-DTF. A summary of the characteristics of these studies is provided in Table 1.

**Table 1:** Characteristics of included trials (*n* = 6).

Study, year	Sample size	Sex	Mean age (years)	Time after stroke	Intervention	Outcome measures
Cho et al. 2018 (11)	E: 12 C: 13	E: male/female: 7/5 C:male/female:6/7	E: 47.58 ± 13.00 C: 52.53 ± 9.06	E: 174.41 ± 117.15 (days) C: 190.30 ± 226.79 (days)	E: IMT 3 sets (30 repetitions/set), 1-min of rest after each set. 5 times/week, for 6 weeks. IMT with a load of 30% of MIP (adjusted every weeks, according the new MIP value). Device: PowerBreath-K5. RHB program as C. C: Conventional stroke physical therapy program (60 min, 5 times/week, for 6 weeks).	A-DTI A-DTE NA-DTE NA-DTF
Lee et al. 2019 (20)	E: 13 C: 12	E: male/female: 7/6 C: male/female: 5/7	E:58.62 ± 12.38 C: 59.75 ± 13.38	E: 11.15 ± 2.38 (months) C: 11.0 ± 2.17 (months)	E: IMT/EMT 5 set for 20 min (10–15 repetitions/set), 30–60 s of rest after each set. 3 times/week, for 6 weeks. IMT/EMT with a load of 30% of IMT/EMT (adjusted every weeks, according the new IMT/EMT value). Device: Threshold PEP, Threshold IMT-Philips Respironics, Andover, MA. Trunk stabilisation exercise(20 min, 3 times/week, for 6 weeks). RHB program as C. C: Conventional stroke physical therapy program (30 min, 2 times/day, 6 times/week, for 6 weeks). Trunk stabilisation exercise(40 min, 3 times/week, for 6 weeks).	A-DTI A-DTE NA-DTE NA-DTF
Wang et al. 2020 (21)	E: 12 C: 12	E: male/female: 7/5 C: male/female: 9/3	E: 57.83 ± 12.62 C: 64.25 ± 14.01	E: 48.25 ± 15.74 (days) C: 40.08 ± 25.98 (days)	E: IMT 3 set for 20 min (10 repetitions/set), 2-min of rest after each set. 5 times/week, for 4 weeks. Load of IMT starting at a load of 30% MIP. It was gradually increased, 5–10% each session, to 60% of MIP as tolerated. Device: PowerBreath-K5. RHB program as C. C: Conventional stroke physical therapy program (5 times/week, for 4 weeks).	A-DTI A-DTE A-DTF A-DM
Kılıçoğlu et al. 2022 (9)	E: 20 C: 21	E: male/female: 10/10 C: male/female: 8/13	E: 64.6 ± 12.4 C: 66 ± 10.3	E: 134.3 ± 163.0 (days) C: 133.8 ± 246.0 (days)	E: IMT/EMT 45 min, 5 times/week, for 6 weeks. Device: Incentive spirometer. RHB program as C. C: Conventional stroke physical therapy program (60 min, 5 times/week, for 6 weeks).	A-DTI A-DTE NA-DTF NA-DTI
Dang et al. 2023 (22)	E: 51 C: 51	E: male/female: 29/22 C: male/female: 27/24	E: 52.36 ± 5.65 C: 52.29 ± 5.61	E: 40.12 ± 5.69 (days) C: 40.08 ± 5.77 (days)	E: IMT 3 set for 20 min (10 repetitions/set), 2-min of rest after each set. 5times/week, for 4 weeks. Load of IMT starting at a load of 30% MIP. It was gradually increased, 5–10% each session,to 60% of MIP as tolerated. Device: PowerBreath-K5. RHB program as C. C: Conventional stroke physical therapy program (60 min, 5 times/week, for 6 weeks).	A-DM
Jung et al. 2013 (23)	E:15 C:14	E: male/female: 7/8 C: male/female: 10/4	E: 58.66 ± 8.39 C:59.64 ± 10.96	E: >6 months C: >6 months	E: IMT 20 min, 3 times/week, for 6 weeks. Load of IMT starting at a load of 30% MIP. (adjusted every week). Device: Threshold inspiratory muscle. RHB program as C. C: Conventional stroke physical therapy program (for 6 weeks)	A-DTI A-DTE NA-DTE NA-DTF

E, experimental group; C, control group; MIP, maximal inspiratory pressure; RHB, rehabilitation; IMT, inspiratory muscle training; EMT, expiratory muscle training; A-DTI, affected diaphragm thickness at inspiration; A-DTE, affected diaphragm thickness at expiration; NA-DTI, non-affected diaphragm thickness at inspiration; NA-DTE, non-affected diaphragm thickness at expiration; A-DTF, affected diaphragm thickening fraction; NA-DTF, non-affected diaphragm thickening fraction; A-DM, affected diaphragm mobility.

### 3.3 Assessment of methodological quality and risk of bias across studies

The mean PEDro score among the included trials was 6. All of the included studies reported random assignment and differences between groups, point estimates and variability. The highest score – 7 points – was achieved by 1 study (11). Four studies (9, 20, 21, 23) scored 6 points, primarily due to the challenge or impossibility of blinding therapists and participants in this type of intervention. The lowest score – 5 points – was assigned to 1 study (22). However, none of the studies reported intention-to-treat analyses, and none of them blinded the therapists. Consequently, 6 studies (9, 20–23) obtained ≥5 points, a moderate quality (Table 2). The results of the risk of bias assessment can be found in Supplementary Appendix 2. In general, blinding of patients and therapists was not feasible due to the nature of the intervention, and most studies lost points due to a lack of allocation concealment and blinding of subjects and therapists.

**Table 2:** PEDro criteria and scores for the included papers (*n* = 6).

Study	Q1	Q2	Q3	Q4	Q5	Q6	Q7	Q8	Q9	Q10	Q11	Total	Study quality
Cho et al. 2018 (11)	Yes	Yes	Yes	Yes	Yes	No	Yes	No	No	Yes	Yes	7	Good
Lee et al. 2019 (20)	Yes	Yes	Yes	Yes	No	No	Yes	No	No	Yes	Yes	6	Good
Kılıçoğlu et al. 2022 (9)	Yes	Yes	No	Yes	No	No	Yes	Yes	No	Yes	Yes	6	Good
Wang et al. 2020 (9)	Yes	Yes	No	Yes	No	No	Yes	Yes	No	Yes	Yes	6	Good
Dang et al. 2023 (9)	Yes	Yes	No	Yes	No	No	No	Yes	No	Yes	Yes	5	Acceptable
Jung et al. 2013 (23)	Yes	Yes	No	Yes	No	No	Yes	Yes	No	Yes	Yes	6	Good

## 4 Synthesis of results

### 4.1 Affected side diaphragm mobility

**Figure 2:**
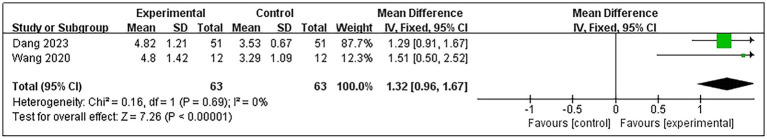
Forest plot for A-DM.

Regarding patients’ A-DM, 2 studies were included (21, 22), with a total of 126 post-stroke patients, including 63 in the experimental group and 63 in the control group. Heterogeneity testing indicated no significant heterogeneity among these studies (*p* > 0.1 and *I*^2^ = 0%); thus, the fixed-effects model was employed. The meta-analysis results revealed that RMT significantly improved A-DM in post-stroke patients compared with the control group (MD = 1.32, 95% CI: 0.96–1.67; *p* < 0.00001) (Figure 2).

### 4.2 Affected side diaphragm thickness at inspiration

**Figure 3:**
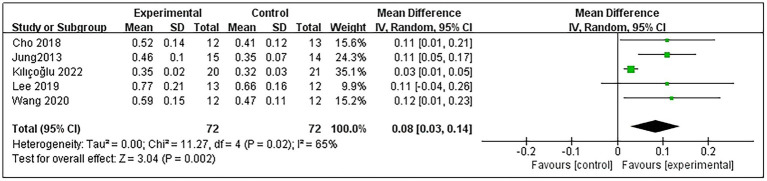
Forest plot for A-DTI.

Concerning patients’ A-DTI, 5 studies were included (9, 11, 20, 21, 23), involving a total of 144 post-stroke patients, with 72 in the experimental group and 72 in the control group. Heterogeneity testing indicated significant heterogeneity among these studies (*p* = 0.02 and *I*^2^ = 65%); thus, the random-effects model was used. The meta-analysis results showed that RMT significantly improved A-DTI in post-stroke patients compared with the control group (MD = 0.08, 95% CI: 0.03–0.14; *p* = 0.002) (Figure 3). Subgroup analyses were conducted based on intervention duration, with 4 studies (9, 11, 20, 23) implementing a 6-week intervention and 1 study (21) lasting 4 weeks. Subgroup analysis demonstrated that RMT, whether for 6 or 4 weeks, significantly improved A-DTI in post-stroke patients (MD = 0.08, 95% CI: 0.02–0.14; *p* = 0.01 and MD = 0.12, 95% CI: 0.01–0.23; *p* = 0.03). Detailed subgroup analysis results are presented in Supplementary Appendix 3.

### 4.3 Affected side diaphragm thickness at expiration

**Figure 4:**
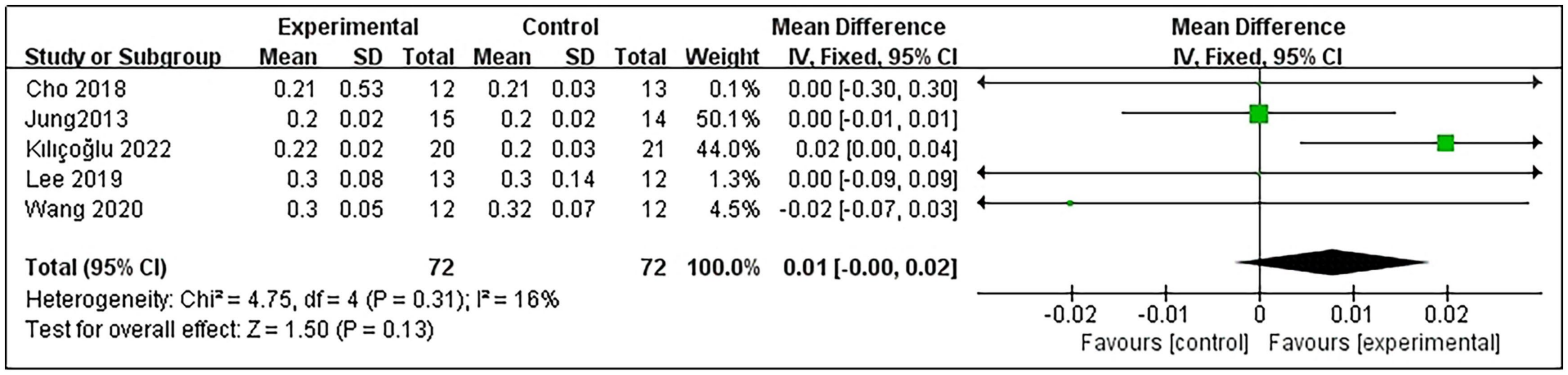
Forest plot for A-DTE.

Concerning patients’ A-DTE, 5 studies were included (9, 11, 20, 21, 23), involving a total of 144 post-stroke patients, with 72 in the experimental group and 72 in the control group. Heterogeneity testing indicated no significant heterogeneity among these studies (*p* = 0.31 and *I*^2^ = 16%); thus, the fixed-effects model was employed. The meta-analysis results demonstrated that RMT did not significantly improve A-DTE in post-stroke patients compared with the control group (MD = 0.01, 95% CI: 0.00–0.02; *p* = 0.13) (Figure 4). Subgroup analyses showed that for the duration of the intervention, neither 6 weeks of RMT (9, 11, 20, 23) nor 4 weeks of RMT (21) significantly improved A-DTE in post-stroke patients (MD = 0.01, 95% CI: 0.00–0.02; *p* = 0.09 and MD = −0.02, 95% CI: −0.07–0.03; *p* = 0.42). Detailed subgroup analysis results are presented in Supplementary Appendix 3.

### 4.4 Non-affected side diaphragm thickness at inspiration

**Figure 5:**
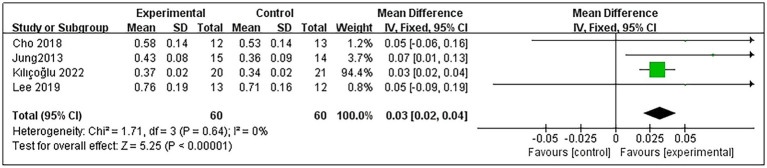
Forest plot for NA-DTI.

Regarding patients’ NA-DTI, 4 studies were included (9, 11, 20, 23), encompassing a total of 120 post-stroke patients, with 60 in the experimental group and 60 in the control group. Heterogeneity testing indicated no significant heterogeneity among these studies (*p* = 0.64 and *I*^2^ = 0%); thus, the fixed-effects model was used. The meta-analysis results revealed that RMT significantly improved NA-DTI in post-stroke patients compared with the control group (MD = 0.03, 95% CI: 0.02–0.04; *p* < 0.00001) (Figure 5).

### 4.5 Non-affected side diaphragm thickness at expiration

**Figure 6:**
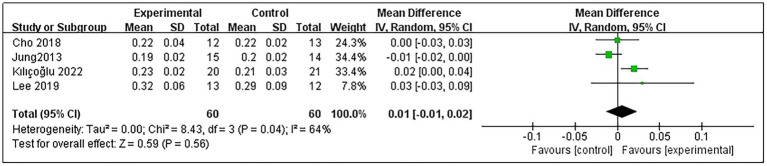
Forest plot for NA-DTE.

Regarding patients’ NA-DTE, 4 studies were included (9, 11, 20, 23), comprising a total of 120 post-stroke patients, with 60 in the experimental group and 60 in the control group. Heterogeneity testing indicated no significant heterogeneity among these studies (*p* = 0.04 and *I*^2^ = 64%); thus, the random-effects model was used. The meta-analysis results demonstrated that RMT did not significantly improve NA-DTE in post-stroke patients compared with the control group (MD = 0.01, 95% CI: −0.01–0.02; *p* = 0.56) (Figure 6).

### 4.6 Affected side diaphragm thickening fraction

**Figure 7:**
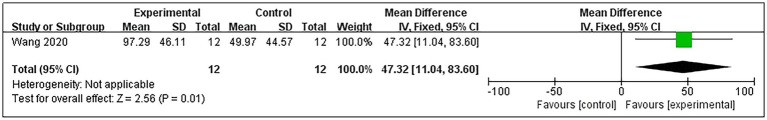
Forest plot for A-DTF.

Only 1 study (21) reported A-DTF in post-stroke patients. The study results indicated that RMT significantly improved A-DTF (MD = 47.32, 95% CI: 11.04–83.60; *p* = 0.01) (Figure 7).

### 4.7 Non-affected side diaphragm thickening fraction

**Figure 8:**
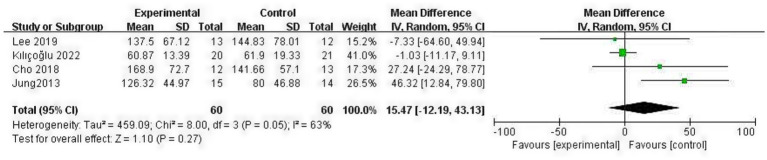
Forest plot for NA-DTF.

Data sufficient to calculate the non-A-DTF (NA-DTF) were available from 4 studies (9, 11, 20, 23), involving a total of 120 post-stroke patients (60 in each group). Heterogeneity testing indicated moderate heterogeneity among these studies (*p* = 0.05, *I*^2^ = 63%); thus, a random-effects model was used. The meta-analysis demonstrated that RMT did not significantly improve NA-DTF in post-stroke patients compared with the control group (MD = 15.47, 95% CI: −12.19–43.13; *p* = 0.27) (Figure 8).

## 5 Discussion

This systematic review found that RMT improved diaphragm function, including A-DM, A-DTI and A-DF, but was ineffective for DT at expiration (DTE). We propose that RMT enhances diaphragm function in post-stroke patients, which in turn may lead to improvements in pulmonary ventilation, respiratory muscle strength, balance and motor function. Therefore, implementing RMT during the early stages of stroke is recommended to prevent diaphragm atrophy and enhance diaphragm function, with the potential to subsequently improve a range of other patient functions. However, it remains uncertain whether these improvements translate into enhanced activity and participation.

Although the beneficial effects of RMT on conventional pulmonary function parameters in stroke patients are well-established, the rationale for specifically assessing diaphragm function as a distinct outcome measure warrants clarification. Pulmonary function tests (e.g., spirometry) represent composite measures influenced by multiple factors, including lung compliance, airway resistance and the coordinated effort of various respiratory muscles. In contrast, direct ultrasonographic measurement of diaphragm morphology (thickness) and contractile performance (excursion, thickening fraction) provides a targeted evaluation of the primary muscle of inspiration. This focused approach allows us to isolate and confirm the specific structural and functional adaptations of the diaphragm itself resulting from RMT, thereby moving beyond correlative improvements to establish a more direct mechanistic link between the intervention and its physiological effects. Furthermore, given the diaphragm’s vital role in postural control, quantifying its function separately helps elucidate its contribution to critical non-respiratory outcomes, such as balance and trunk stability, in the stroke population.

Regarding A-DM in post-stroke patients, the meta-analysis results demonstrated a significant enhancement in A-DM in post-stroke patients following RMT. It is worth noting that prior studies have also found that RMT can notably improve pulmonary ventilation function in stroke patients (12–14), and there is a significant positive correlation between diaphragm mobility (DM) and pulmonary ventilation function (8). For every 1 cm drop in the diaphragm, pulmonary ventilation increases by 250–350 mL (24). The research results of Geun et al. (25) indicate that A-DM in stroke patients decreases by an average of 21.4% compared with normal individuals during quiet breathing, and 1 study (26) suggests that the decrease in cough flow in patients is related to DM. Stroke reduced respiratory muscle strength and cough flow by approximately 50% in patients, and MIP decreased by 41.39 cm H_2_O compared with normal (7, 10). Respiratory muscle weakness, decreased pulmonary ventilation and cough effectiveness lead to secretion retention, making the risk of stroke-associated pneumonia (SAP) much higher. Menezes et al. (27) conducted a systematic review of the correlation between RMT and SAP, confirming that RMT reduces the risk of respiratory system complications in patients after stroke, and an improvement in DM may be one of the important reasons.

Concerning DT in post-stroke patients, the meta-analysis indicated that RMT increases significantly for DT at inspiration (DTI) but has no benefit for DTE. This is similar to the results of a meta-analysis conducted by Fabero-Garrido et al. (15). The conclusions of this review showed that RMT significantly improved exercise tolerance, respiratory muscle function, DT and lung function in the short term; however, none of these effects were preserved in the medium term. The lack of significant improvement in DTE may be attributed to several factors. First, the limited number of studies – only 4 randomised controlled trials of moderate methodological quality – constrains the robustness of this finding. Second, expiration primarily engages the abdominal muscles, with comparatively less contribution from the diaphragm. Furthermore, previous evidence suggests that RMT may have limited efficacy in specifically strengthening expiratory muscles in this patient population (7). Previous studies have found that diaphragm function is significantly reduced not only on the hemiplegic side in post-stroke patients but also on the non-hemiplegic side, with an asymmetric increase in DT, leading to asymmetric diaphragm contraction and impaired lung function (2, 28–30). In one study, Khedr et al. (6) found an abnormal magnetic potential latency and central conduction time in the cortex of the affected side of the brain in post-stroke patients, and the diaphragm to central conduction time on the hemiplegic side was completely missing or significantly prolonged. Researchers using transcranial magnetic stimulation, concentrating on an area 3 cm lateral to the midline and 2–3 cm anterior to the auricular plane, have observed that the diaphragm responds to stimulation of a single cerebral hemisphere predominantly contralateral to the hemisphere, with a lower ipsilateral response (31). Voyvoda et al. (32) similarly concluded that the diaphragm has both contralateral (predominantly contralateral innervation) and ipsilateral innervation and that the innervation shows marked individual differences. The results of a meta-analysis by Zhang et al. (33) showed a significant increase in DT on the hemiplegic side of stroke patients with IMT, whereas no change was observed on the non-hemiplegic side. However, the results of the present meta-analysis showed that RMT improved DT on both sides of the diaphragm in post-stroke patients, but the improvement was more significant in the A-DTI than in the NA-DTI; this improved the asymmetry of DT on both sides at the same time, which is in agreement with the conclusions of previous studies (2, 15). This finding suggests that RMT may be an effective intervention for mitigating asymmetry in DT. The observed bilateral increase in DT following RMT may result from physiological adaptations aligned with the overload principle, whereby application of resistance induces an increase in the diaphragmatic muscle cross-sectional area (34). The focus of our consideration of RMT is to enhance A-DTI. With respect to A-DTI, which involved a total of 5 studies (9, 11, 20, 21, 23), the heterogeneity test results initially indicated significant heterogeneity. A sensitivity analysis was performed by sequentially excluding studies, and it was observed that the heterogeneity reduced from 50 to 0% after removing the study by Kılıçoğlu et al. (9). This suggests that the primary source of heterogeneity stemmed from the study by Kılıçoğlu et al. The reasons for the initial heterogeneity may be attributed to several factors. First, the study did not clearly describe the methods of concealed allocation. Second, although RMT was utilised in the study, it did not employ a threshold loading device, unlike the other 4 studies that used similar devices. Notably, traditional RMT, including abdominal breathing and chest expansion training, was employed by Kılıçoğlu et al. (9), whereas the remaining 4 studies (11, 20, 21, 23) utilised threshold loading devices. Our analysis indicated that interventions utilising threshold loading devices produced more pronounced improvements in A-DTI than those employing traditional RMT techniques. Therefore, we considered that RMT with a threshold loading device yields more substantial improvements in A-DTI in post-stroke patients. The detailed results of sensitivity analyses are shown in Supplementary Appendix 4.

Regarding the DTF, this study analysed the affected (A-DTF) and non-affected (NA-DTF) sides separately. The results indicated that RMT significantly improved A-DTF in post-stroke patients, but the improvement in NA-DTF did not reach statistical significance. Although a direct statistical comparison between sides was not feasible due to A-DTF data being from a single study, the difference in effect sizes (A-DTF: MD = 47.32; NA-DTF: MD = 15.47) suggests that the improvement in contractile function of the affected hemidiaphragm following RMT may be more pronounced than that of the non-affected side. This finding aligns with the pattern observed in DT changes (i.e., greater improvement in A-DTI than in NA-DTI) and further supports the notion that RMT may help correct the common post-stroke asymmetry in diaphragmatic function by preferentially enhancing the function and structure of the affected side. The lack of significant improvement in NA-DTF could be attributed to the relatively preserved neural drive and baseline function of the non-affected hemidiaphragm, leaving less room for improvement, or to the limited sample size and statistical power in the present analysis. Future prospective studies directly reporting bilateral DTF are needed to confirm this observation.

Previous research has demonstrated the beneficial effects of RMT on lung function, respiratory muscle strength, walking ability and trunk stability in stroke patients (12–15, 27, 35–37). However, few studies have concurrently investigated changes in diaphragmatic function. Given that the diaphragm is a primary respiratory muscle responsible for up to 70% of ventilation activity and plays a crucial role in trunk stability, we hypothesised that RMT may enhance lung function, respiratory muscle strength, walking ability and trunk stability by improving diaphragmatic function. Correlations between DT and pulmonary ventilation function and balance function have been reported (5, 8, 35, 38). In a study by Cohn et al. (39), they found that during respiratory activity, DT increased linearly as lung volume increased and increased more rapidly as total lung capacity was approached. The diaphragm was more effective in increasing muscle thickness during deep inspiration than during quiet breathing, and DT and muscle function were more closely related during contraction than at rest; DT was positively correlated with MIP and lung capacity (39, 40). Jung et al. (8) found a relationship between respiratory function and DT and diaphragm excursion, especially on the hemiplegic side of the diaphragm. Therefore, the role of the diaphragm on the hemiplegic side is important in rehabilitation projects to improve respiratory function in stroke patients. The recovery of the patient’s diaphragm function can improve the patient’s lung function, which is beneficial to the rehabilitation of motor function, and the improvement of motor function improves the patient’s lung function, forming a benign cycle.

Trunk muscle weakness in post-stroke patients, as well as loss of trunk position sense, disrupts trunk control in stroke patients and negatively affects postural control. Studies have shown that 96.4% of stroke patients have trunk impairments, and 83% of acute stroke patients have problems with balance and postural control, which is one of the main causes of disability in stroke patients (41, 42). Hodges and Gandevia (43) noted that the diaphragm has a stability function, acting indirectly by increasing intra-abdominal pressure and supporting the spine and directly by sustained co-contraction, which contributes to postural stability; thus, impaired diaphragmatic activation in post-stroke patients leads to reduced trunk stability, which is closely related to normal balance and mobility. Kocjan et al. (35) found that the diaphragm plays an important role in maintaining the stability of trunk posture and that reduced diaphragm muscle thickness and restricted movement were strongly associated with balance dysfunction. Similar to these findings, Liu et al. (5) also found that diaphragm function was positively correlated with limb motor and balance function on the hemiplegic side. The results of numerous studies (36, 37, 44, 45) have shown that RMT interventions can effectively improve balance function and trunk control in stroke patients, and Aydoğan Arslan et al. (36) suggested that RMT during the acute phase may have a more significant effect on postural control and sitting balance. Nevertheless, a meta-analysis by Pozuelo-Carrascosa et al. (46) indicated that although RMT intervention in stroke patients positively correlated with functional abilities, such as walking capacity (i.e., the 6-Minute Walk Test), it did not significantly affect balance. This discrepancy could be attributed to the limited sample size of the study by Pozuelo-Carrascosa et al. (45), highlighting the need for more extensive, high-quality studies to investigate whether the benefits of RMT extend to exercise and participation in post-stroke patients.

Regarding DTF in post-stroke patients, our analysis included only 1 study (21), leading to a descriptive analysis. Nonetheless, it was found that RMT effectively improved A-DTF in post-stroke patients. Both DT and DTF serve as indicators of actual diaphragm strength in patients, akin to the ‘ejection fraction’ principle of the heart (47). The increase in inspiratory muscle strength during RMT, attributed to increased strength of the inspiratory muscles during contraction, likely involves an increase in DT during contraction. Lower DTF values, reflecting diaphragmatic activity and its work during respiration, are associated with greater homeostatic deficits, with values <20% suggesting diaphragmatic atrophy (35). Liu et al. (48) found that the rate of diaphragmatic thickening on the hemiplegic side of stroke patients was negatively correlated with the duration of the disease, and the longer the duration of stroke was, the greater the likelihood that the patient had varying degrees of diaphragmatic atrophy. Early stroke patients did not show symptoms of dyspnoea because (1) lung capacity is nonlinearly related to muscle strength, so the reduction in lung capacity occurs later in the development of muscle dysfunction (49), and (2) in patients after stroke, who separate respiratory effort from dyspnoea, dyspnoea is perceived to be low and delayed (46). It is worth noting that although the diaphragm acts as a skeletal muscle, it does atrophy eight times faster than skeletal muscle (50). Although early stroke patients do not have symptoms of dyspnoea, early RMT to prevent diaphragmatic atrophy is essential.

Systematic reviews have previously demonstrated that RMT significantly enhances pulmonary ventilation function and respiratory muscle strength in post-stroke patients (4, 7, 27). The results of this meta-analysis further support the notion that RMT significantly improves diaphragm function in post-stroke patients. Given the significant correlations between DT, DM, pulmonary ventilation function and respiratory muscle strength, it is reasonable to suggest that RMT may improve pulmonary ventilation and respiratory muscle strength by enhancing diaphragm function. Consequently, larger clinical trials are warranted to investigate whether RMT does improve pulmonary ventilation function and respiratory muscle strength due to improved diaphragm function.

Regarding the optimal duration of RMT, a previous meta-analysis concluded that 30 min of RMT, five times per week for 5 weeks, was sufficient to increase respiratory muscle strength in post-stroke patients (27). However, a specific treatment duration for improving diaphragm function after stroke has not been reported. Given the limited number of studies included in this meta-analysis, we recommend a minimum of 4 weeks of RMT training to improve diaphragm function. A publication bias analysis was not conducted due to the inclusion of <10 studies.

### 5.1 Limitations

Although this meta-analysis suggests that RMT improves diaphragm function in post-stroke patients, there are several limitations to consider. First, the small sample size of the included studies may introduce bias into the results. Second, variations in patient characteristics and the nature of respiratory training interventions could lead to differences in outcomes. Third, the included studies had differences in measurement techniques for DT, which may have contributed to the variability in results. Fourth, this study solely explored the impact of RMT on diaphragm function in post-stroke patients and did not investigate other outcome measures, such as pulmonary function or exercise endurance. The researchers emphasise the potential of RMT to enhance diaphragm function, particularly A-DTI. Therefore, future meta-analyses should broaden their focus to assess the multifaceted effects of RMT on post-stroke patients. Additionally, this study did not delve into the long-term effects of RMT on diaphragm function in post-stroke patients, leaving the question of whether RMT has enduring effects on the diaphragm unanswered. Consequently, it is essential to explore the long-term impacts of RMT on diaphragm function in post-stroke patients and evaluate the effects of various prescribed RMT modes on endurance during physical activity and overall quality of life in future clinical investigations.

## 6 Conclusion

This meta-analysis holds clinical significance for rehabilitation practise. Respiratory muscle training shows promise in enhancing diaphragmatic function in post-stroke patients, potentially serving as a valuable component of post-stroke treatment. However, given the limitations, such as the small sample size and study quality, it is imperative to incorporate larger-scale, higher-quality studies to further investigate the effectiveness of RMT in post-stroke patients.

## Data Availability

The original contributions presented in the study are included in the article/Supplementary material, further inquiries can be directed to the corresponding authors.

## References

[1] Lozano R Naghavi M Foreman K Lim S Shibuya K Aboyans V . Global and regional mortality from 235 causes of death for 20 age groups in 1990 and 2010: a systematic analysis for the global burden of disease study 2010. Lancet. (2012) 380:2095–128. doi: 10.1016/S0140-6736(12)61728-0, 23245604 PMC10790329

[2] Jung J Kim N. The effect of progressive high-intensity inspiratory muscle training and fixed high-intensity inspiratory muscle training on the asymmetry of diaphragm thickness in stroke patients. J Phys Ther Sci. (2015) 27:3267–9. doi: 10.1589/jpts.27.3267, 26644689 PMC4668180

[3] Catalá-Ripoll JV Monsalve-Naharro JÁ Hernández-Fernández F. Incidence and predictive factors of diaphragmatic dysfunction in acute stroke. BMC Neurol. (2020) 20:79. doi: 10.1186/s12883-020-01664-w, 32138697 PMC7057624

[4] Martín-Valero R De La Casa AM Casuso-Holgado MJ Heredia-Madrazo A. Systematic review of inspiratory muscle training after cerebrovascular accident. Respir Care. (2015) 60:1652–9. doi: 10.4187/respcare.03981, 26493591

[5] Liu X Qu Q Deng P Zhao Y Liu C Fu C . Assessment of diaphragm in hemiplegic patients after stroke with ultrasound and its correlation of extremity motor and balance function. Brain Sci. (2022) 12:882. doi: 10.3390/brainsci12070882, 35884689 PMC9313444

[6] Khedr EM El Shinawy O Khedr T Abdel Aziz Ali Y Awad EM. Assessment of corticodiaphragmatic pathway and pulmonary function in acute ischemic stroke patients. Eur J Neurol. (2000) 7:509–16. doi: 10.1046/j.1468-1331.2000.00104.x, 11054135

[7] Pollock RD Rafferty GF Moxham J Kalra L. Respiratory muscle strength and training in stroke and neurology: a systematic review. Int J Stroke. (2013) 8:124–30. doi: 10.1111/j.1747-4949.2012.00811.x, 22568454

[8] Jung JH Kim NS. The correlation between diaphragm thickness, diaphragmatic excursion, and pulmonary function in patients with chronic stroke. J Phys Ther Sci. (2017) 29:2176–9. doi: 10.1589/jpts.29.2176, 29643599 PMC5890225

[9] Kılıçoğlu MS Yurdakul OV Çelik Y Aydın T. Investigating the correlation between pulmonary function tests and ultrasonographic diaphragm measurements and the effects of respiratory exercises on these parameters in hemiplegic patients. Top Stroke Rehabil. (2022) 29:218–29. doi: 10.1080/10749357.2021.1911748, 33844946

[10] Kulnik ST. Should we train respiratory muscles after stroke? Neurology. (2015) 85:560–1. doi: 10.1212/WNL.0000000000001846, 26180138

[11] Cho J Lee H Kim M Lee W. The improvement in respiratory function by inspiratory muscle training is due to structural muscle changes in patients with stroke: a randomized controlled pilot trial. Top Stroke Rehabil. (2018) 25:37–43. doi: 10.1080/10749357.2017.1383681, 29061084

[12] Jung KM Bang DH. Effect of inspiratory muscle training on respiratory capacity and walking ability with subacute stroke patients: a randomized controlled pilot trial. J Phys Ther Sci. (2017) 29:336–9. doi: 10.1589/jpts.29.336, 28265169 PMC5333000

[13] Britto RR Rezende NR Marinho KC Torres JL Parreira VF Teixeira-Salmela LF. Inspiratory muscular training in chronic stroke survivors: a randomized controlled trial. Arch Phys Med Rehabil. (2011) 92:184–90. doi: 10.1016/j.apmr.2010.09.029, 21272713

[14] Messaggi-Sartor M Guillen-Solà A Depolo M Duarte E Rodríguez DA Barrera MC . Inspiratory and expiratory muscle training in subacute stroke: a randomized clinical trial. Neurology. (2015) 85:564–72. doi: 10.1212/WNL.0000000000001827, 26180145

[15] Fabero-Garrido R Del Corral T Angulo-Díaz-Parreño S Plaza-Manzano G Martín-Casas P Cleland JA . Respiratory muscle training improves exercise tolerance and respiratory muscle function/structure post-stroke at short term: a systematic review and meta-analysis. Ann Phys Rehabil Med. (2022) 65:101596. doi: 10.1016/j.rehab.2021.101596, 34687960

[16] Moher D Shamseer L Clarke M Ghersi D Liberati A Petticrew M . Preferred reporting items for systematic review and meta-analysis protocols (PRISMA-P) 2015 statement. Syst Rev. (2015) 4:1. doi: 10.1186/2046-4053-4-1, 25554246 PMC4320440

[17] Higgins JP Altman DG Gøtzsche PC Jüni P Moher D Oxman AD . The Cochrane collaboration's tool for assessing risk of bias in randomised trials. BMJ. (2011) 343:d5928. doi: 10.1136/bmj.d5928 22008217 PMC3196245

[18] Bhogal SK Teasell RW Foley NC Speechley MR. The PEDro scale provides a more comprehensive measure of methodological quality than the Jadad scale in stroke rehabilitation literature. J Clin Epidemiol. (2005) 58:668–73. doi: 10.1016/j.jclinepi.2005.01.002, 15939217

[19] Foley NC Teasell RW Bhogal SK Speechley MR. Stroke rehabilitation evidence-based review: methodology. Top Stroke Rehabil. (2003) 10:1–7. doi: 10.1310/Y6TG-1KQ9-LEDQ-64L8, 12970828

[20] Lee K Park D Lee G. Progressive respiratory muscle training for improving trunk stability in chronic stroke survivors: a pilot randomized controlled trial. J Stroke Cerebrovasc Dis. (2019) 28:1200–11. doi: 10.1016/j.jstrokecerebrovasdis.2019.01.008, 30712955

[21] Lu W Yihui C Xiu Z Linlin L Xiao L. The effect of inspiratory muscle training on pulmonary function and diaphragm movement after stroke. Chin J Phys Med Rehabil. (2020) 42:6. doi: 10.3760/cma.j.issn.0254-1424.2020.11.006

[22] Dang H Xiaofei L Weirong C Jianming L Feifei Y. Effects of inspiratory muscle training combined with conventional rehabilitation training on diaphragm movement and pulmonary function of stroke patients. J Zhengzhou Univ Med Sci. (2023) 2:241–6. doi: 10.13705/j.issn.1671-6825.2022.06.079

[23] Jung J Kim N. Effects of inspiratory muscle training on diaphragm thickness, pulmonary function, and chest expansion in chronic stroke patients. J Korean Soc Phys Med. (2013) 8:59–69. doi: 10.13066/kspm.2013.8.1.059

[24] Haotian Z Yaru Y Yi L Hongyuan X. Diaphragm, intercostal muscle thickening fraction and intercostal muscle compensatory index for evaluating respiratory muscle function in elderly patients with mechanical ventilation. Chin J Med Imaging Technol. (2021) 37:28. doi: 10.13929/j.issn.1003-3289.2021.09.028

[25] Park GY Kim SR Kim YW Jo KW Lee EJ Kim YM . Decreased diaphragm excursion in stroke patients with dysphagia as assessed by M-mode sonography. Arch Phys Med Rehabil. (2015) 96:114–21. doi: 10.1016/j.apmr.2014.08.019, 25234476

[26] Choi YM Park GY Yoo Y Sohn D Jang Y Im S. Reduced diaphragm excursion during reflexive citric acid cough test in subjects with subacute stroke. Respir Care. (2017) 62:1571–81. doi: 10.4187/respcare.05488, 28900040

[27] Menezes KK Nascimento LR Ada L Polese JC Avelino PR Teixeira-Salmela LF. Respiratory muscle training increases respiratory muscle strength and reduces respiratory complications after stroke: a systematic review. J Physiother. (2016) 62:138–44. doi: 10.1016/j.jphys.2016.05.014, 27320833

[28] Kim N Jung J. The effects of breathing retraining on asymmetry of diaphragm thickness in stroke patients. J Korean Soc Phys Med. (2013) 8:263–9. doi: 10.13066/kspm.2013.8.2.263

[29] Sutbeyaz ST Koseoglu F Inan L Coskun O. Respiratory muscle training improves cardiopulmonary function and exercise tolerance in subjects with subacute stroke: a randomized controlled trial. Clin Rehabil. (2010) 24:240–50. doi: 10.1177/0269215509358932, 20156979

[30] Houston JG Morris AD Grosset DG Lees KR McMillan N Bone I. Ultrasonic evaluation of movement of the diaphragm after acute cerebral infarction. J Neurol Neurosurg Psychiatry. (1995) 58:738–41. doi: 10.1136/jnnp.58.6.738, 7608679 PMC1073558

[31] Similowski T Catala M Rancurel G Derenne JP. Impairment of central motor conduction to the diaphragm in stroke. Am J Respir Crit Care Med. (1996) 154:436–41. doi: 10.1164/ajrccm.154.2.8756819, 8756819

[32] Voyvoda N Yücel C Karatas G Oguzülgen I Oktar S. An evaluation of diaphragmatic movements in hemiplegic patients. Br J Radiol. (2012) 85:411–4. doi: 10.1259/bjr/71968119, 21712430 PMC3485549

[33] Zhang X Zheng Y Dang Y Wang L Cheng Y Zhang X . Can inspiratory muscle training benefit patients after stroke? A systematic review and meta-analysis of randomized controlled trials. Clin Rehabil. (2020) 34:866–76. doi: 10.1177/0269215520926227, 32493056

[34] McCool FD Benditt JO Conomos P Anderson L Sherman CB Hoppin FG. Variability of diaphragm structure among healthy individuals. Am J Respir Crit Care Med. (1997) 155:1323–8. doi: 10.1164/ajrccm.155.4.9105074, 9105074

[35] Kocjan J Gzik-Zroska B Nowakowska K Burkacki M Suchoń S Michnik R . Impact of diaphragm function parameters on balance maintenance. PLoS One. (2018) 13:e0208697. doi: 10.1371/journal.pone.0208697, 30592726 PMC6310257

[36] Aydoğan Arslan S Uğurlu K Sakizli Erdal E Keskin ED Demirgüç A. Effects of inspiratory muscle training on respiratory muscle strength, trunk control, balance and functional capacity in stroke patients: a single-blinded randomized controlled study. Top Stroke Rehabil. (2022) 29:40–8. doi: 10.1080/10749357.2020.1871282, 33412997

[37] Pai HC Li CC. Relationship between trunk control ability and respiratory function in stroke patients: a scoping review and Meta-analysis. Asian Nurs Res (Korean Soc Nurs Sci). (2023) 17:61–9. doi: 10.1016/j.anr.2023.04.001, 37080388

[38] Kim M Lee K Cho J Lee W. Diaphragm thickness and inspiratory muscle functions in chronic stroke patients. Med Sci Monit. (2017) 23:1247–53. doi: 10.12659/MSM.900529, 28284044 PMC5358861

[39] Cohn D Benditt JO Eveloff S McCool FD. Diaphragm thickening during inspiration. J Appl Physiol (1985). (1997) 83:291–6. doi: 10.1152/jappl.1997.83.1.291, 9216975

[40] McCool FD Conomos P Benditt JO Cohn D Sherman CB Hoppin FG. Maximal inspiratory pressures and dimensions of the diaphragm. Am J Respir Crit Care Med. (1997) 155:1329–34. doi: 10.1164/ajrccm.155.4.9105075, 9105075

[41] Kong KH Ratha KR. Truncal impairment after stroke: clinical correlates, outcome and impact on ambulatory and functional outcomes after rehabilitation. Singapore Med J. (2021) 62:87–91. doi: 10.11622/smedj.2019153, 31788705 PMC8027135

[42] Tyson SF Hanley M Chillala J Selley A Tallis RC. Balance disability after stroke. Phys Ther. (2006) 86:30–8. doi: 10.1093/ptj/86.1.30, 16386060

[43] Hodges PW Gandevia SC. Activation of the human diaphragm during a repetitive postural task. J Physiol. (2000) 522:165–75. doi: 10.1111/j.1469-7793.2000.t01-1-00165.x, 10618161 PMC2269747

[44] Lee K Cho JE Hwang DY Lee W. Decreased respiratory muscle function is associated with impaired trunk balance among chronic stroke patients: a Cross-sectional study. Tohoku J Exp Med. (2018) 245:79–88. doi: 10.1620/tjem.245.79, 29848898

[45] Janssens L McConnell AK Pijnenburg M Claeys K Goossens N Lysens R . Inspiratory muscle training affects proprioceptive use and low back pain. Med Sci Sports Exerc. (2015) 47:12–9. doi: 10.1249/MSS.0000000000000385, 24870567

[46] Pozuelo-Carrascosa DP Carmona-Torres JM Laredo-Aguilera JA Latorre-Román PÁ Párraga-Montilla JA Cobo-Cuenca AI. Effectiveness of respiratory muscle training for pulmonary function and walking ability in patients with stroke: a systematic review with Meta-analysis. Int J Environ Res Public Health. (2020) 17:5356. doi: 10.3390/ijerph17155356, 32722338 PMC7432552

[47] Dinino E Gartman EJ Sethi JM McCool FD. Diaphragm ultrasound as a predictor of successful extubation from mechanical ventilation. Thorax. (2014) 69:431–5. doi: 10.1136/thoraxjnl-2013-204111, 24365607

[48] Xiaoman L Ying Y Qingming Q Panmo D Yuehua Z Chenghong L. Analysis of diaphragmatic function and the related factors in stroke patients. Chin J Stroke. (2022) 17:579–84. doi: 10.3969/j.issn.1673-5765.2022.06.004

[49] Sferrazza Papa GF Pellegrino GM Di Marco F Imeri G Brochard L Goligher E . A review of the ultrasound assessment of diaphragmatic function in clinical practice. Respiration. (2016) 91:403–11. doi: 10.1159/000446518, 27216909

[50] Thomason DB Biggs RB Booth FW. Protein metabolism and beta-myosin heavy-chain mRNA in unweighted soleus muscle. Am J Phys. (1989) 257:R300–5. doi: 10.1152/ajpregu.1989.257.2.R300, 2764153

