# Supplementary material for: Effect of respiratory muscle training on diaphragm function in stroke patients: a systematic review and meta-analysis
Source: Front Med (Lausanne). 2026 Jan 20;12:1694356. doi: 10.3389/fmed.2025.1694356 (PMC12866612; doi:10.3389/fmed.2025.1694356)
Supplement: Supplementary file 1 [file Table_1.DOCX]

# Supplemental Appendix 1

**Databases:CKNI, Wan Fang Data, Pubmed, EMBASE, PEDro, Web of Science, clinical trials.gov, The Cochrane Library**

**The Cochrane Library**

#1.MeSH descriptor: [Stroke] explode all trees

#2.(Strokes):ti, ab, kw OR (Cerebrovascular Accident):ti, ab, kw OR (Cerebrovascular Accidents):ti, ab, kw OR (CVA (Cerebrovascular Accident)):ti, ab, kw OR (CVAs (Cerebrovascular Accident)):ti, ab, kw (Word variations have been searched)

#3.(Cerebrovascular Apoplexy):ti, ab, kw OR (Apoplexy, Cerebrovascular):ti, ab, kw OR (Vascular Accident, Brain):ti, ab, kw OR (Brain Vascular Accident):ti, ab, kw OR (Brain Vascular Accidents):ti, ab, kw (Word variations have been searched)

#4.(Apoplexy):ti, ab, kw OR (Cerebral Stroke):ti, ab, kw OR (Cerebrovascular Strokes):ti, ab, kw OR (Cerebral Strokes):ti, ab, kw OR (Stroke, Cerebral):ti, ab, kw (Word variations have been searched)

#5.(Strokes, Cerebral):ti, ab, kw OR (Stroke, Acute):ti, ab, kw OR (Acute Stroke):ti, ab, kw OR (Acute Strokes):ti, ab, kw OR (Strokes, Acute):ti, ab, kw (Word variations have been searched)

#6.(Cerebrovascular Accident, Acute):ti, ab, kw OR (Acute Cerebrovascular Accident):ti, ab, kw OR (Acute Cerebrovascular Accidents):ti, ab, kw OR (Cerebrovascular Accidents, Acute):ti, ab, kw (Word variations have been searched)

#7.#1 or #2 or #3 or #4 or #5 or #6

#8.MeSH descriptor: [Breathing Exercises] explode all trees

#9.(Exercise, Breathing):ti, ab, kw OR (Respiratory Muscle Training):ti, ab, kw OR (Muscle Training, Respiratory):ti, ab, kw OR (Training, Respiratory Muscle):ti, ab, kw (Word variations have been searched)

#10 .#8 or #9

#12 .#7 and #10

**EMBASE**

#1. 'stroke'/exp

#2. 'strokes':ab, kw, ti OR 'cerebrovascular accident':ab, kw, ti OR 'cerebrovascular accidents':ab, kw, ti OR 'cva (cerebrovascular accident)':ab, kw, ti OR' cvas (cerebrovascular accident)':ab, kw, ti OR 'cerebrovascular apoplexy':ab, kw, ti OR 'apoplexy, cerebrovascular':ab, kw, ti OR 'vascular accident, brain':ab, kw, ti OR 'brain vascular accident':ab, kw, ti OR 'brain vascular accidents':ab, kw, ti OR 'vascular accidents, brain':ab, kw, ti OR 'cerebrovascular stroke':ab, kw, ti OR 'cerebrovascular strokes':ab, kw, ti OR 'stroke, cerebrovascular':ab, kw, ti OR 'strokes, cerebrovascular':ab, kw, ti OR 'apoplexy':ab, kw, ti OR 'cerebral stroke':ab, kw, ti OR 'cerebral strokes':ab, kw, ti OR 'stroke, cerebral':ab, kw, ti OR 'strokes, cerebral':ab, kw, ti OR 'stroke, acute':ab, kw, ti OR 'acute stroke':ab, kw, ti OR 'acute strokes':ab, kw, ti OR 'strokes, acute':ab, kw, ti OR 'cerebrovascular accident, acute':ab, kw, ti OR 'acute cerebrovascular accident':ab, kw, ti OR 'acute cerebrovascular accidents':ab, kw, ti OR 'cerebrovascular accidents, acute':ab, kw, ti

#3. #1 OR #2

#4. 'breathing exercise'/exp OR 'breathing exercise'

#5. 'exercise, breathing':ab, kw, ti OR 'respiratory muscle training':ab, kw, ti OR 'muscle training, respiratory':ab, kw, ti OR 'training, respiratory muscle':ab, kw, ti

#6. 'random':ab, ti OR 'placebo':ab, ti OR 'double-blind':ab, ti

#7. #4 OR #5

#8. #3 AND #6 AND #7

**Pubmed**

("randomized controlled trial"[Publication Type] OR "randomized"[Title/Abstract] OR "placebo"[Title/Abstract]) AND ("Breathing Exercises"[MeSH Terms] OR ("exercise breathing"[Title/Abstract] OR "respiratory muscle training"[Title/Abstract] OR "muscle training respiratory"[Title/Abstract] OR "training respiratory muscle"[Title/Abstract])) AND ("Strokes"[Title/Abstract] OR "cerebrovascular accident"[Title/Abstract] OR "cerebrovascular accidents"[Title/Abstract] OR (("Stroke"[MeSH Terms] OR "Stroke"[All Fields] OR "cva"[All Fields]) AND "cerebrovascular accident"[Title/Abstract]) OR "cerebrovascular accidents acute"[Title/Abstract] OR "acute cerebrovascular accidents"[Title/Abstract] OR "acute cerebrovascular accident"[Title/Abstract] OR "cerebrovascular accident acute"[Title/Abstract] OR "strokes acute"[Title/Abstract] OR "acute strokes"[Title/Abstract] OR "acute stroke"[Title/Abstract] OR "stroke acute"[Title/Abstract] OR "strokes cerebral"[Title/Abstract] OR "stroke cerebral"[Title/Abstract] OR "cerebral strokes"[Title/Abstract] OR "cerebral stroke"[Title/Abstract] OR "Apoplexy"[Title/Abstract] OR "strokes cerebrovascular"[Title/Abstract] OR "stroke cerebrovascular"[Title/Abstract] OR "cerebrovascular strokes"[Title/Abstract] OR "cerebrovascular stroke"[Title/Abstract] OR (("blood vessels"[MeSH Terms] OR ("blood"[All Fields] AND "vessels"[All Fields]) OR "blood vessels"[All Fields] OR "Vascular"[All Fields] OR "neovascularization, pathologic"[MeSH Terms] OR ("neovascularization"[All Fields] AND "pathologic"[All Fields]) OR "pathologic neovascularization"[All Fields] OR "vascularisation"[All Fields] OR "vascularization"[All Fields] OR "vascularisations"[All Fields] OR "vascularise"[All Fields] OR "vascularised"[All Fields] OR "vascularities"[All Fields] OR "vascularitis"[All Fields] OR "vascularity"[All Fields] OR "vascularizations"[All Fields] OR "vascularize"[All Fields] OR "vascularized"[All Fields] OR "vascularizes"[All Fields] OR "vascularizing"[All Fields] OR "vasculars"[All Fields]) AND "accidents brain"[Title/Abstract]) OR "brain vascular accidents"[Title/Abstract] OR "brain vascular accident"[Title/Abstract] OR "vascular accident brain"[Title/Abstract] OR "apoplexy cerebrovascular"[Title/Abstract] OR "cerebrovascular apoplexy"[Title/Abstract] OR "Stroke"[MeSH Terms])

**Web of Science**

TS=(Strokes OR Cerebrovascular Accident OR Cerebrovascular Accidents OR CVA OR CVAs OR Cerebrovascular Apoplexy OR Brain Vascular Accident OR Brain Vascular Accidents OR Cerebrovascular Stroke OR Cerebrovascular Strokes OR Apoplexy OR Cerebral Stroke OR Cerebral Strokes OR Acute Stroke OR Acute Strokes OR Acute Cerebrovascular Accident OR Acute Cerebrovascular Accidents) AND TS=(Breathing Exercises or Exercise, Breathing OR Respiratory Muscle Training OR Muscle Training, Respiratory OR Training , Respiratory Muscle) AND TS=(randomized controlled trial OR randomized OR placebo)

**PEDro**

Abstract & Title: Stroke

Therapy: respiratory therapy

Problem: no selection

Body part: no selection

Subdiscipline: no selection

Method: Clinical Trial

**Clinical trials.gov**

Condition or disease :Stroke

Study type: Interventional Studies（Clinical Trials）

Study Results:Studies With Results

Age :All

Sex :All

Intervention/treatment: Respiratory Muscle Training OR Breathing Exercises OR respiratory therapy

**CNKI**

(((SU= stroke + cerebral infarction + hemorrhagic stroke + cerebral hemorrhage + Ischemic Stroke OR TI= stroke + cerebral infarction + hemorrhagic stroke + cerebral hemorrhage + Ischemic Stroke) OR (SU= cerebrovascular accident + Acute stroke + Apoplexy OR TI= cerebrovascular accident + Acute stroke + Apoplexy)) AND (SU= Respiratory Muscle Training + Breathing Exercises OR TI= Respiratory Muscle Training + Breathing Exercises))

**Wan Fang Data**

((SU=(stroke OR cerebral infarction OR hemorrhagic stroke OR cerebral hemorrhage OR Ischemic Stroke OR cerebrovascular accident OR Acute stroke OR Apoplexy) AND (SU=Respiratory Muscle Training OR Breathing Exercises) AND (SU=randomized)

# Supplemental Appendix 2

**Figure. 1** Summary of risk of bias for included studies


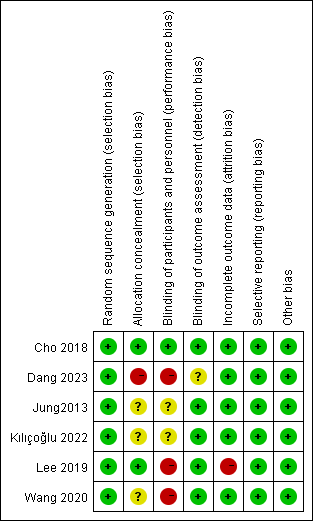


**Figure. 2** Graph of risk of bias for included studies


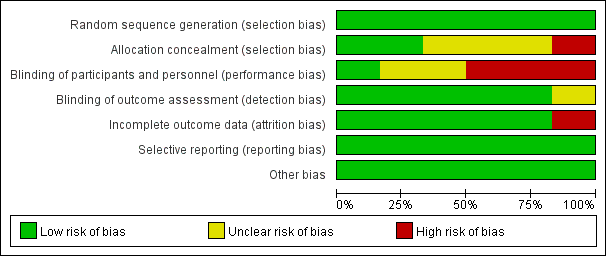


# Supplemental Appendix 3

**Table 1** Results of subgroup analyses

| Outcome indicators | Category | Number of included studies | MD（95% CI） | Heterogeneity test results (P value and I^2)^ | Overall effect (Z value and P value) |
| --- | --- | --- | --- | --- | --- |
| Affected side diaphragm thickness at inspiration（A-DTI） | Intervention time | | | | |
|  | Six weeks | 4 | 0.08 (0.02，0.14) | P=0.03  I^2^=66% | Z=2.59  P=0.01 |
|  | Four weeks | 1 | 0.12 (0.01, 0.23) | - | Z=2.23  P=0.03 |
| Affected side diaphragm thickness at expiration（A-DTE） | Intervention time | | | | |
|  | Six weeks | 4 | 0.01 (0.00, 0.02) | P=0.03  I^2^=13% | Z=1.71  P=0.09 |
|  | Four weeks | 1 | -0.02 (-0.07, 0.03) | - | Z=0.81  P=0.42 |

# Supplemental Appendix 4

**Table 2** Results of sensitivity analyses

| Outcome indicators | Study of removal | MD（95% CI） | Heterogeneity test results (P value and I^2^ value) | Overall effect (Z value and P value) |
| --- | --- | --- | --- | --- |
| Affected side diaphragm thickness at inspiration（A-DTI） | After removing Cho, 2018 | 0.08 (0.02, 0.14) | P=0.02  I^2^=68% | Z=2.58  P=0.01 |
|  | After removing Kılıçoğlu, 2022 | 0.11 (0.07, 0.16) | P=1.00  I^2^=0% | Z=4.84  P＜0.00001 |
|  | After removing Lee, 2019 | 0.08 (0.02, 0.14) | P=0.02  I^2^=71% | Z=2.71  P=0.007 |
|  | After removing Wang, 2020 | 0.08 (0.02, 0.14) | P=0.03  I^2^=66% | Z=2.59  P=0.01 |
|  | After removing  Jung, 2013 | 0.07 (0.01, 0.13) | P=0.11  I^2^=50% | Z=2.45  P=0.01 |
| Affected side diaphragm thickness at expiration（A-DTE） | After removing Cho, 2018 | 0.01 (0.00, 0.02) | P=0.19  I^2^=37% | Z=1.50  P=0.13 |
|  | After removing Kılıçoğlu, 2022 | 0.02 (-0.02, 0.01) | P=0.90  I^2^=0% | Z=0.23  P=0.82 |
|  | After removing Lee, 2019 | 0.01 (0.00, 0.02) | P=0.19  I^2^=36% | Z=1.51  P=0.13 |
|  | After removing Wang, 2020 | 0.01 (0.00, 0.02) | P=0.33  I^2^=13% | Z=1.71  P=0.09 |
|  | After removing  Jung, 2013 | 0.02 (0.00, 0.03) | P=0.48  I^2^=0% | Z=2.13  P=0.03 |
| Non-affected side diaphragm thickness at inspiration（NA-DTI） | After removing Cho, 2018 | 0.03 (0.02, 0.04) | P=0.45  I^2^=0% | Z=5.18  P＜0.00001 |
|  | After removing Kılıçoğlu, 2022 | 0.06(0.01, 0.11) | P=0.93  I^2^=0% | Z=2.46  P=0.01 |
|  | After removing Lee, 2019 | 0.03 (0.02, 0.04) | P=0.44  I^2^=0% | Z=5.20  P＜0.00001 |
|  | After removing  Jung, 2013 | 0.03 (0.02, 0.04) | P=0.90  I^2^=0% | Z=4.91  P＜0.00001 |
|  |  |  |  |  |
| Non-affected side diaphragm thickness at expiration（NA-DTE）） | After removing Cho, 2018 | 0.01 (-0.02, 0.03) | P=0.02  I^2^=76% | Z=0.62  P=0.53 |
|  | After removing Kılıçoğlu, 2022 | -0.01 (-0.02, 0.01) | P=0.39  I^2^=0% | Z=0.94  P=0.35 |
|  | After removing Lee, 2019 | 0.00 (-0.02, 0.02) | P=0.02  I^2^=74% | Z=0.34  P=0.73 |
|  | After removing  Jung, 2013 | 0.02 (0.00, 0.03) | P=0.37  I^2^=0% | Z=2.30  P=0.02 |
